# Berlin as a Medical Center

**Published:** 1885-07

**Authors:** 


**Berlin as a Medical Center.** A Guide for American practitioners and students, by H. R. Bigelow, M. D., &c., Sandy Hook, Conn. New England Publishing Co. 1885.

As the title indicates, this little book is simply a guide for medical practitioners and students in Berlin. Those of our readers who contemplate study in Germany, should by all means obtain it. We know from personal observation that without some such aid as this, valuable time is wasted in finding out about the courses, time and place of classes, etc. A study of this little book will enable the student to get into good working routine in Berlin without any trouble, and at a great saving of expense as well as time.

